# The Staff Mental Health Service in Cambridgeshire and Peterborough: a new model for assessment and treatment of healthcare workers in the context of COVID-19 pandemic

**DOI:** 10.1192/bjo.2021.142

**Published:** 2021-06-18

**Authors:** Muzaffer Kaser, Zoe Martin, Cathy Walsh

**Affiliations:** 1 Cambridgeshire and Peterborough NHS foundation trust, Department of Psychiatry, University of Cambrige; 2 Cambridgeshire and Peterborough NHS foundation trust, Cambridge University Hospitals NHS Foundation Trust; 3 Cambridgeshire and Peterborough NHS foundation trust

## Abstract

**Aims:**

Staff mental health is a major determinant of a well-functioning health system that has become ever more important during the COVID-19 pandemic. Poor mental health is the most common reason for NHS staff sickness absences, usually accounting for 25% of all reported sick leave. At a time when the NHS most needs an available and efficient workforce, government and NHS employers lack the necessary evidence to inform decisions about how best to support the mental health needs of its staff. In this report, we share our experience and the initial figures from a newly developed multidisciplinary assessment and treatment service for NHS staff.

**Method:**

The Staff Mental Health Service (SMHS) at the Cambridgeshire and Peterborough NHS Foundation Trust (CPFT) launched in September 2020. The SMHS is commissioned by the Cambridgeshire and Peterborough sustainability and transformation partnership and is accessible to the 25,513 staff based at five NHS trusts within the region. The service received 235 referrals within 5 months of the launch. All patients had a first clinical contact within three working days and more than 80% had their initial assessment within two weeks. The SMHS clinical team is comprised of consultant psychiatrists, senior clinical psychologists, specialist mental health nurses, and an occupational health nurse set to provide rapid access, confidential, evidence-based treatments for the NHS staff. As part of service evaluation within CPFT, we collected routine screening data (Patient Health Questionnaire-9 (PHQ-9), Generalised Anxiety Disorder-7 (GAD-7), and Posttraumatic Symptom Check List – 6 (PCL-6)) from patients completing the initial assessment (n = 130).

**Result:**

According to the initial figures (n = 130) from a diverse group of healthcare staff, on average the patients presented with moderate level of depressive symptoms (PHQ-9: 16.22 ± 5.94). Anxiety levels were in moderate to severe range (GAD-7: 13.45 ± 4.70). Average score of PCL-6 checklist for traumatic stress symptoms was higher than the established cut-off (>14): 19.43 ± 5.65.

**Conclusion:**

The Staff Mental Health Service offers an innovative, multi-disciplinary rapid assessment and treatment clinic for NHS staff. The demand for the service has been immense, reaching double the number of predicted referrals. Initial data suggested high rates of moderate to severe depression, anxiety, and traumatic stress symptoms in healthcare workers. Our clinical observations was that many healthcare workers have had longstanding significant mental health conditions that saw deterioration during the COVID-19 pandemic. We hope that our experience in the SMHS will help inform models across the UK to address the clear unmet need for staff mental health.

